# Supplementary material for: Cortical reactivation of spatial and non-spatial features coordinates with hippocampus to form a memory dialogue
Source: Nat Commun. 2023 Nov 27;14:7748. doi: 10.1038/s41467-023-43254-7 (PMC10682454; doi:10.1038/s41467-023-43254-7)
Supplement: Supplementary file 1 — Supplementary Information [file 41467_2023_43254_MOESM1_ESM.pdf]

Supplementary Information for

Cortical Reactivation of Spatial and Non-Spatial Features Coordinates with Hippocampus to Form a Memory Dialogue

HaoRan Chang[1*], Ingrid M. Esteves[1], Adam R. Neumann[1], Majid H. Mohajerani[1, 2], and Bruce L. McNaughton[1, 3]

[1] Canadian Centre for Behavioural Neuroscience, Department of Neuroscience, University of Lethbridge, 4401 University Drive, Lethbridge, T1K 3M4, Alberta, Canada.

[2] Department of Psychiatry, Douglas Hospital Research Centre, McGill University, 6875 Boulevard LaSalle, Verdun, QC, H4H 1R3, Canada.

[3] Department of Neurobiology and Behavior, University of California, 2205 McGaugh Hall, Irvine, 92697, California, United States of America.

* Corresponding author(s). E-mail(s): haoran.chang@mail.mcgill.ca

Contents

Supplementary Figures **3**

Supplementary Tables **31**

Supplementary Methods **32**
- Estimating proportions of cue/spatial cells 32
- Temporal Compression 32
- Modelling cue- and position-correlated responses 33
- Hopfield network modelling 34
- Topographic Organisation 35

List of Supplementary Figures

1 Locomotion entrains slow population dynamics 3
2 REST2 exhibits sparse, synchronous population dynamics 5
3 Hypergeometric modelling of the fraction of spatially-selective cells 6
4 Encoding of visuo-tactile cues is more pronounced following hippocampal lesion . 7
5 Ensemble neurons share neighbouring place fields 9
6 Trajectories consist of short segments frequently spanning cue locations 10
7 Estimated proportions of cue/spatial cells 11
8 Online/offline dynamics of cue and trajectory ensembles 12
9 Temporal compression 13
10 Conjunctive encoding of cue and trajectory representations 14
11 Unsupervised clustering of cue and trajectory ensembles 15
12 SWR timing 17
13 Examples of temporally coupled cue-trajectory ensemble pairs 18
14 Temporally-coupled REST1 ensembles 19
15 Hopfield modelling 20
16 Consecutive recording days 21
17 Persistence of ensemble across days 22
18 Topographic organisation 24
19 ROI registration across days 26
20 Ensemble detection not biased by scan mirrors 27
21 Determining ensemble reactivation strength 28
22 Obtaining the reactivated features 30

List of Supplementary Tables

1 Experimental conditions for individual animal subjects 31

Supplementary Figures

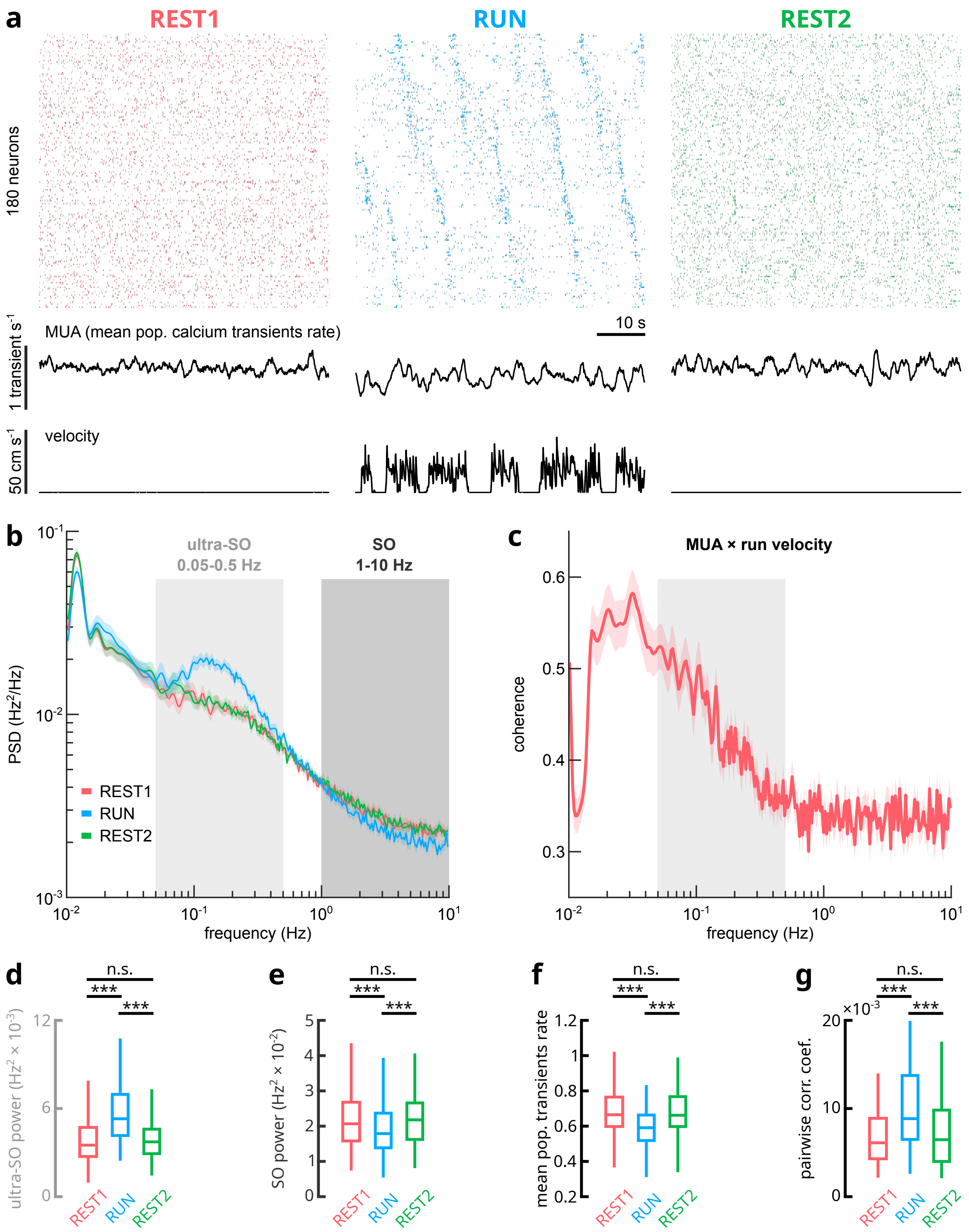

Supplementary Figure 1

Locomotion entrains slow population dynamics. a Binary matrices of calcium events detected by deconvolution of the fluorescent traces. Neurons are sorted by the location of peak activity. 1 min segments from each behavioural epoch are shown (same example session as Fig. 1**c**). The multi-unit activity (MUA), estimated as the rate of transients per neuron averaged over the population at each imaging frame, and the run velocity are illustrated below. **b** Power spectral density (PSD) of the MUA in each behavioural epoch, averaged over all recording sessions ($n = 86$; shaded area denotes $\pm$S.E.M.). PSD was estimated by Welch's method, using 200 s Hann windows with 75% overlap. **c** Coherence between RUN MUA and velocity over the treadmill. Spectral densities were estimated using the same methods as **b**. **d-f** The MUA power in the ultra-slow oscillations (0.05-0.5 Hz) and slow oscillations (1-10 Hz) bands, as well as the mean MUA rates (transients s^{-1}), are compared across behavioural epochs (pairwise Wilcoxon signed rank tests; n.s. $p \geq 0.05$; *** $p < 0.001$; p-values were Bonferroni-adjusted). **g** Pearson correlation coefficients were obtained between the time-series of each pair of neurons within a session, and the average across all pairs was taken. Source data are provided as a Source Data file.

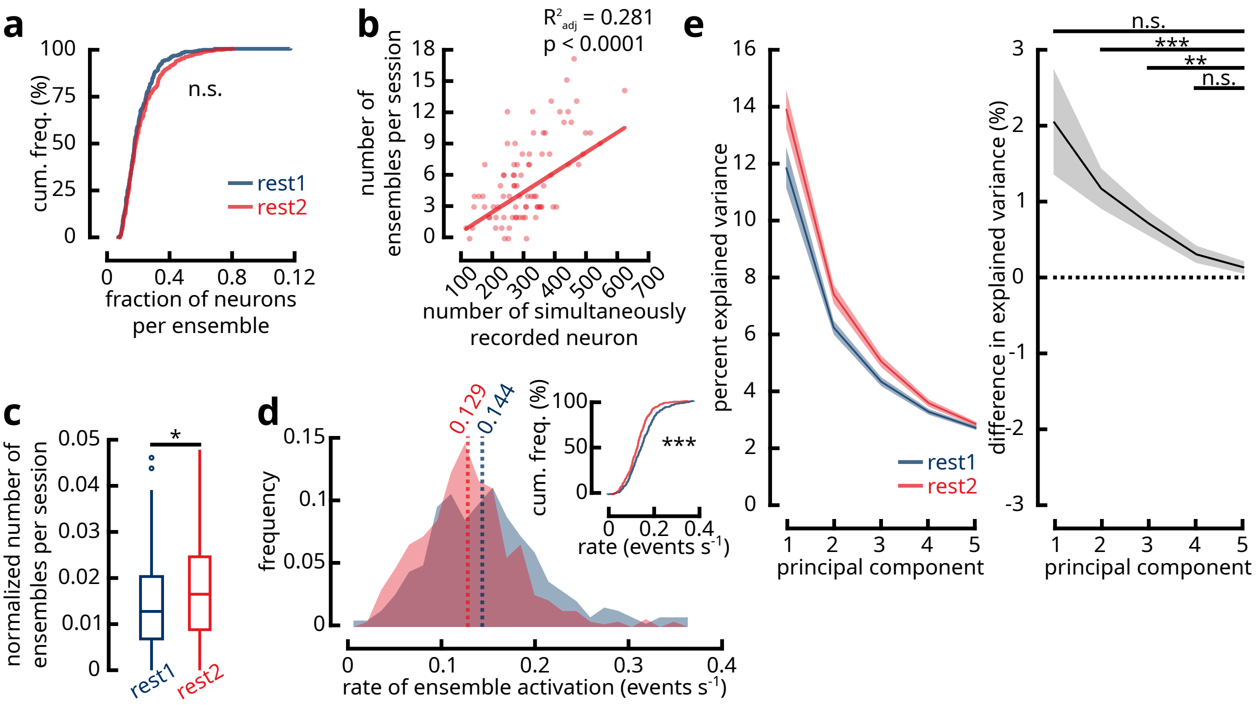

Supplementary Figure 2

REST2 exhibits more synchronous and sparse population dynamics. **a** Cumulative frequency distributions of the fraction of neurons that belong to an ensemble with respect to the total number of simultaneously recorded neurons within a session for REST1 ($n = 86$ sessions; $n = 392$ ensembles) and REST2 ($n = 478$ ensembles) (two-sample two-tailed Kolmogorov–Smirnov test; $p = 0.074$). **b** Number of ensembles per recording session vs. the total number of simultaneously acquired neurons for REST2 ($n = 86$ sessions). **c** Given that the number of ensembles within a recording is dependent upon the total number of recorded neurons, the former quantity needs to be normalized for appropriate comparisons. Depicted is the number of ensembles in REST1 and REST2 expressed as a fraction of the total number of neurons. A higher number of synchronous ensembles are found in individual REST2 sessions ($n = 86$ sessions; Paired-samples Wilcoxon signed-rank test $p = 0.001$). **d** Distributions of the average rate of activation of REST1 ($n = 392$) and REST2 ($n = 478$) ensembles. The median activation rate of REST1 ensembles was higher than that of REST2 ensembles (two-tailed Mann-Whitney U-test $p < 0.001$; two-sample two-tailed Kolmogorov-Smirnov test $p < 0.001$). **e** PCA was conducted over the correlation matrices of time series vectors of simultaneously recorded neurons in REST1 and REST2 separately. The first five principal components were extracted and the percentage of the total variance explained by each component was examined (lines show mean±s.e.m.). For these paired samples ($n = 86$ sessions), we took the difference in percentage explained variance between REST2 and REST1 for each component to test whether REST2 components reliably explain more of the variance (one-way repeated measures ANOVA with Greenhouse-Geisser correction; residuals approximately normal; significant difference between components with $p = 0.019$). Post-hoc tests using the fifth component as a baseline reference suggests that the second and third components in REST2 accounted for more of the variance in the population dynamics than their REST1 counterparts (n.s. $p \geq 0.05$; * $p < 0.05$; ** $p < 0.01$; *** $p < 0.001$; p-values were Bonferroni-adjusted). Source data are provided as a Source Data file.

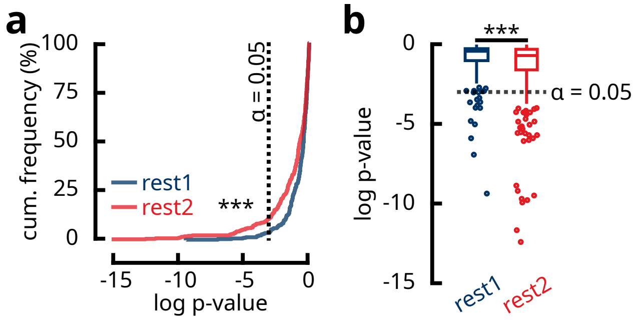

Supplementary Figure 3

Hypergeometric modelling of the fraction of spatially-selective cells in rest ensembles. For each ensemble ($n = 392$ REST1 ensembles; $n = 478$ REST2 ensembles), the number of spatially-selective cells is counted out of the total number of cells that are part of the ensemble. These values are compared to the total number of spatial cells, as well as the total cell count, within the corresponding recording session, using a one-tailed Fisher's exact test. Intuitively, the p-value returned from this test reflects the likelihood of obtaining a certain number of spatial cells in an ensemble of a given size by drawing at chance from the sample population. Small p-values indicate that the fraction of spatial cells within an ensemble is higher than what is expected at chance level. P-values were log-transformed for better detection of low-probability events. **a** Empirical cumulative distribution functions of $\log(p_{\text{values}})$ for REST1 and REST2 ensembles. A greater number of REST2 ensembles contained a large fraction of spatially-selective neurons (two-tailed two-sample Kolmogorov-Smirnov test; $p < 0.001$). Dotted line represents significant α level. **b** Same as **a**, but represented as boxplots (two-tailed Mann-Whitney U-test; $p < 0.001$). Source data are provided as a Source Data file.

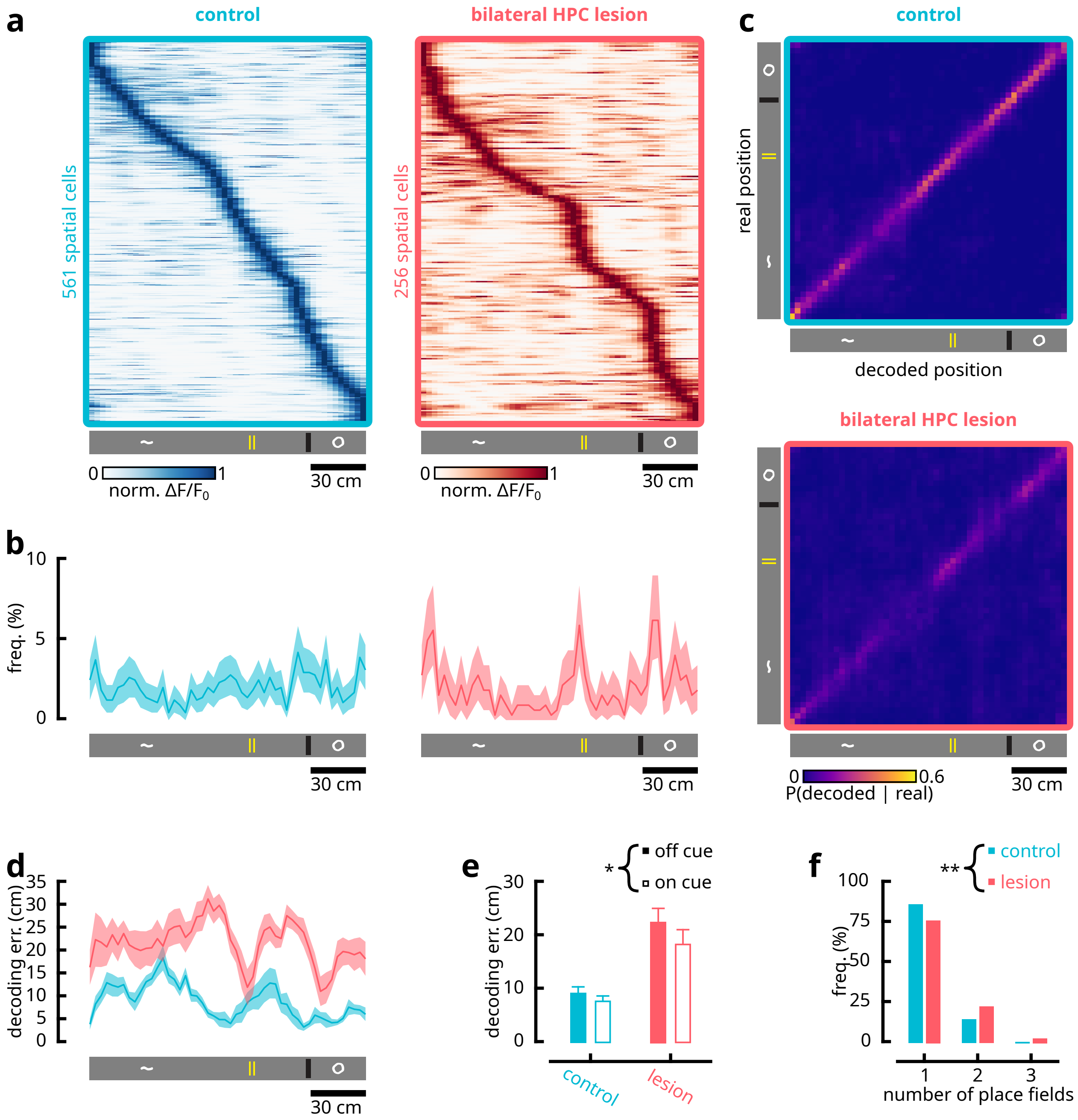

Supplementary Figure 4

Representation of visuo-tactile cues in the secondary motor cortex is more pronounced following bilateral lesion of the dorsal hippocampus. Data from [1]. **a** The average response (normalized between 0 to 1) of spatially-selective neurons as a function of spatial location for control (blue) and hippocampal-lesioned (red) animals. Neurons sorted by their peak average firing location. Note that the population representation of space in control animals is approximately uniform, while this representation is biased towards the locations of cues in the lesioned group. **b** Histogram distribution of place field centres in control (blue; $n = 650$ place fields) and lesioned (red; $n = 327$ place fields) groups. Shaded areas represent 95 % bootstrapped confidence intervals. **c** Normalized confusion matrices for real and Bayesian decoded positions (see [1] for methods). Decoding error

assessed by *leave-one-out* cross-validation over trials. Notice that accuracy is higher over cue locations in the lesion group. **d** Average decoding as a function of spatial location ($n = 10$ sessions in $n = 4$ control animals; $n = 8$ sessions in $n = 4$ lesion animals). Shaded area denote S.E.M. **e** Average decoding error inside and outside cue locations. Robust two-way mixed ANOVA with 20 % trimmed-means (R package 'WRS'). Main effects of hippocampal lesion ($p = 0.010$) and cue location ($p = 0.028$). No significant interaction ($p = 0.080$). **f** Frequency in the number of place fields per individual spatial neurons. Spatial cells in the lesioned group tend to support a higher number of place fields (χ^2 test; $p = 0.001$). Source data are provided as a Source Data file.

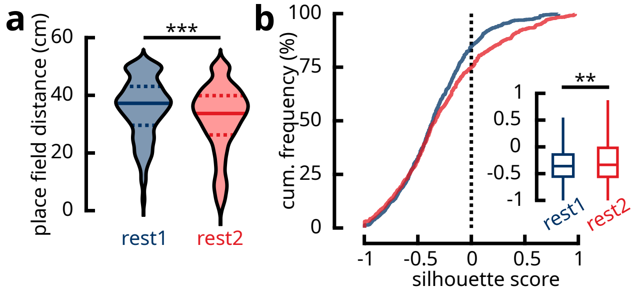

Supplementary Figure 5

REST2 ensembles tend to be composed of spatially-selective neurons sharing neighbouring place fields. a The average distance between place field centres of neurons within the same REST1 or REST2 ensemble ($n = 392$ REST1 ensembles; $n = 478$ REST2 ensembles; $p < 0.001$; Mann-Whitney U-test). The kernel densities for the violin plots were estimated using a 2 cm Gaussian window. The median (solid line), first and last quartiles (dashed lines) are shown. **b** Cumulative distribution functions of the silhouette coefficients of the place field centre locations of REST1 and REST2 neurons that belonged to synchronous ensembles. The silhouette coefficient, in the current context, measures how similar the neurons within the same ensemble are as opposed to neurons outside the ensemble. Similarity is based on the Euclidean distance between place field centres. Silhouette values range from -1 to 1, where high values suggest strong cohesion between a neuron and the other neurons within the same ensemble, and clear separation of the neuron from to the rest of the neuronal population. A significantly larger portion of REST2 neurons contain positive silhouette values compared to REST1 neurons ($n = 1023$ REST1; $n = 1979$ REST2 ensemble neuron place fields; Kolmogorov–Smirnov test $p < 0.001$; Mann-Whitney U-test $p = 0.007$; outliers omitted). Source data are provided as a Source Data file.

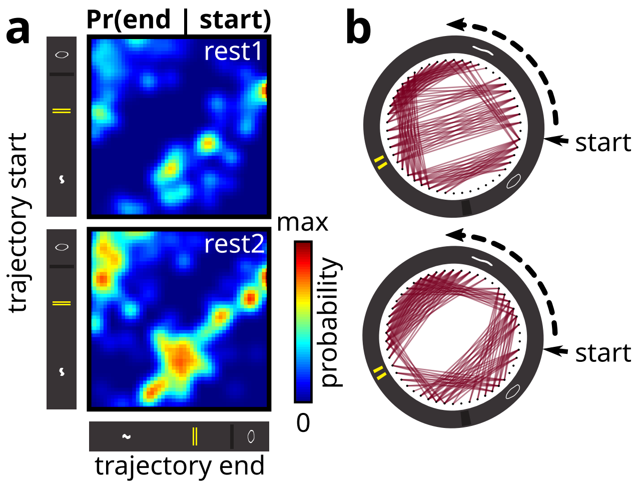

Supplementary Figure 6

Trajectory ensembles encode short spatial segments that tend to span the locations of cues. **a** Conditional probability matrices of the end location of trajectories given their starting location (n = 160 REST1 trajectories; n = 325 REST2 trajectories). Notice that the densities aggregate over a diagonal slightly offset from the central diagonal of the matrix, meaning that the majority the trajectories consist of short segments over space. Within these densities, a large fraction tend to span the locations of cues or the space delimited by two cues. Both of these trends are expressed prominently in REST2 trajectory ensembles, while REST1 ensembles show no clear organization. **b** The same probabilities in **a** represented in graph form. Short arrow point to the beginning of the track. Dashed arrow indicates the running direction.

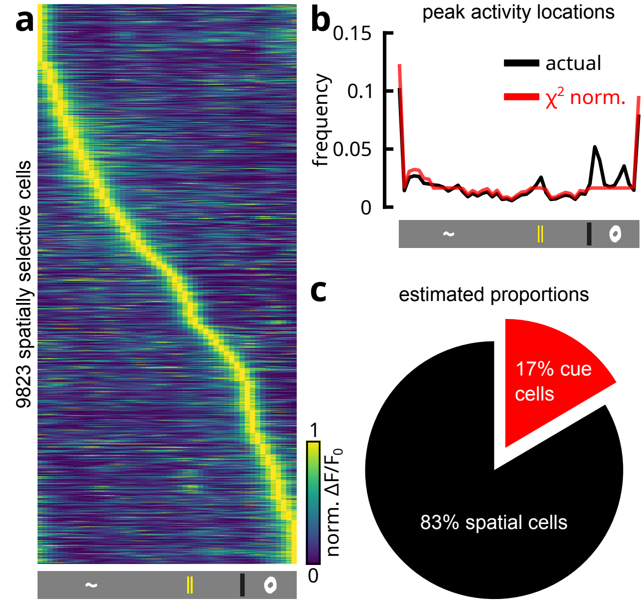

Supplementary Figure 7

Estimating the proportions of cue-responsive cells. **a** The average activity as a function of position for all neurons that passed the criteria for spatial selectivity (all animals; all sessions). The criteria used for identifying these neurons do not discriminate between location-encoding and cue-encoding neurons (see Methods). Assuming that location-encoding cells are uniformly distributed over space, the extra densities observed at cue locations would reflect cells that respond to cue sensation. **b** The distribution of locations of peak activity in **a**. Using a minimum χ^2 estimation procedure (see Supplementary Methods), neurons were iteratively removed at the locations of cues until the distribution was maximally uniform. **c** The fraction of cue-responding cells was estimated by counting the number of cells removed at cue locations in **b**.

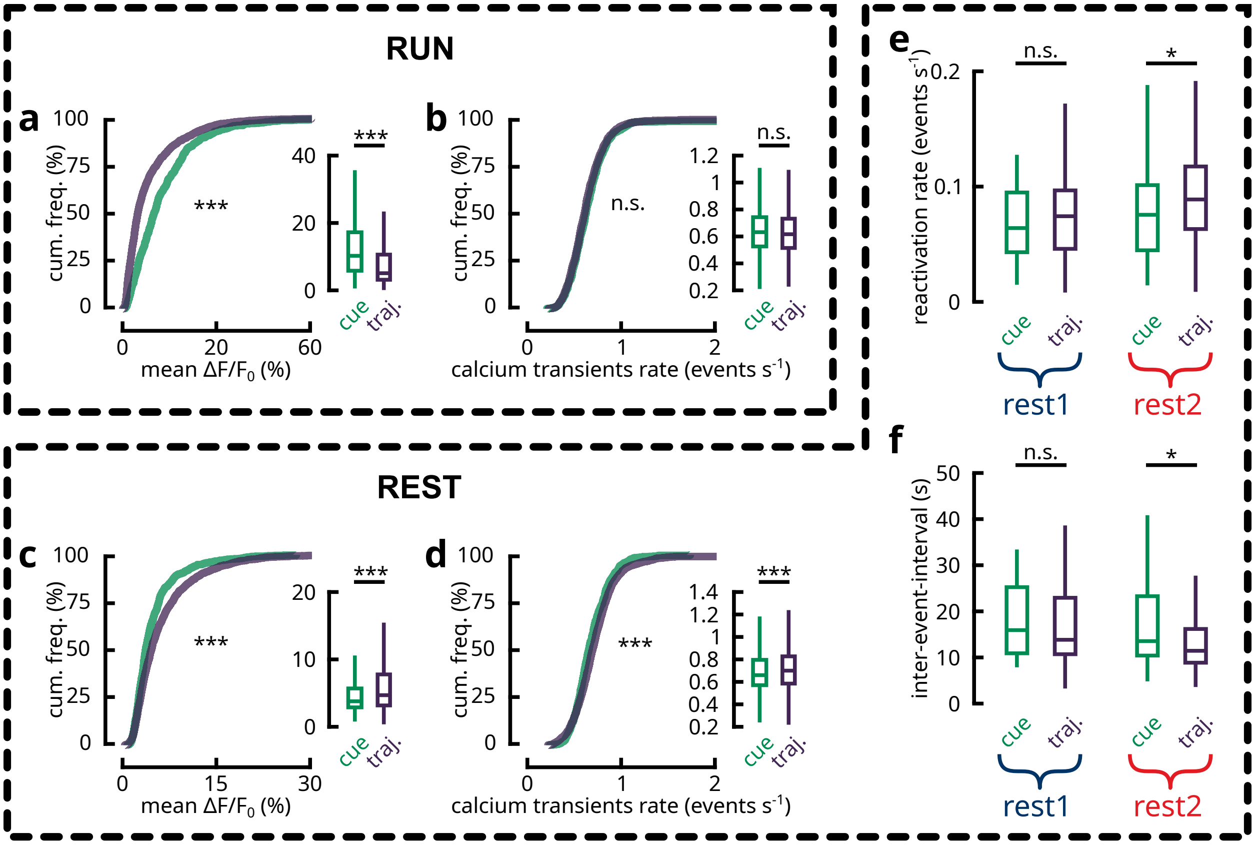

Supplementary Figure 8

Online and offline dynamics of cue and trajectory ensembles. **a-b** Mean $\Delta F/F_0$ and calcium transient rates for cue ($n = 751$) and trajectory ($n = 2056$) ensemble cells during RUN epochs. Cue ensemble neurons exhibit stronger activities than trajectory ensemble neurons in terms of calcium transients, albeit the number of transients per unit time were comparable between the two groups (two-sample two-tailed Kolmogorov-Smirnov test and two-tailed Mann-Whitney U-test; n.s. $p \geq 0.05$; *** $p < 0.001$). **c-d** Same as **a-b**, but during REST epochs. Here, the trends are reversed, whereby trajectory ensemble neurons show stronger activation and slightly more elevated rates of calcium transients. **e-f** Average rate of reactivation events and the inter-event-interval between reactivations for cue ($n = 19$ in REST1; $n = 62$ in REST2) and trajectory ($n = 97$ in REST1; $n = 164$ in REST2) ensembles. Trajectory ensembles reactivate more frequently during REST2 (Mann-Whitney U-test; $p = 0.034$), in accordance with **d**, but at equal rates as cue ensembles during REST1 ($p = 0.732$). Similarly, the average interval between trajectory reactivations was shorter in REST2 (Mann-Whitney U-test; $p = 0.021$), but not during REST1 ($p = 0.704$). All outliers omitted. Source data are provided as a Source Data file.

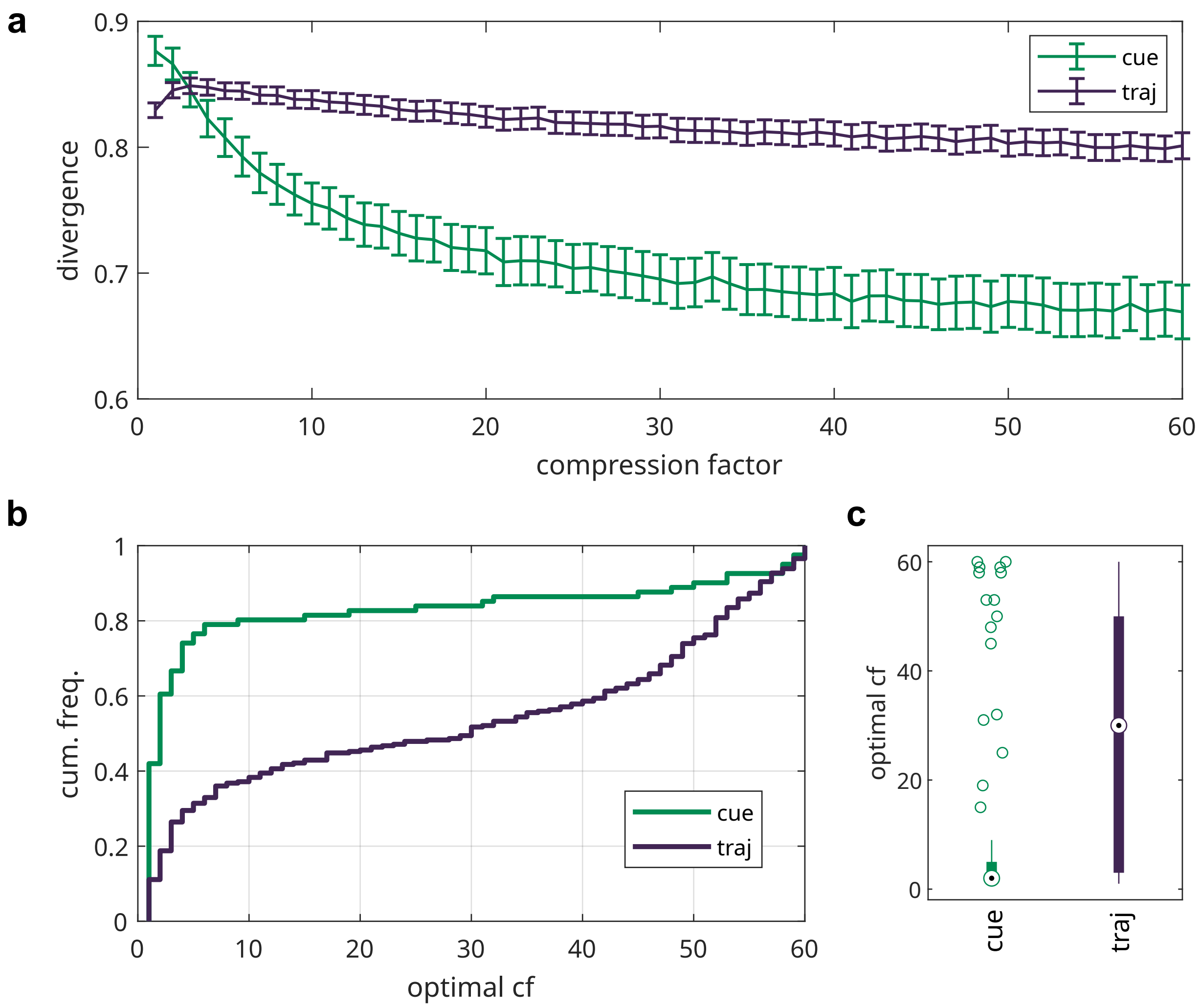

Supplementary Figure 9

Time compressed reactivation of trajectory, but not cue features. **a** Divergence as a function of compression factor for cue ($n = 81$) and trajectory ($n = 261$) ensembles (REST1 and REST2 pooled; mean $\pm$ SEM). Notice that cue ensembles express peak divergence with no compression, while divergence sharply decreases with higher compression. In comparison, trajectory ensembles benefit from some degree of compression. These results are commensurate with the notion that trajectory ensembles contain neurons that firing sequentially during RUN, whereas cue ensemble neurons fire in synchrony during behaviour. **b** Empirical cumulative distribution functions of the compression factor where peak divergence is found for the sample data in **a**. Reactivation of trajectory ensembles is more compressed temporally (median of $30\times$), whereas cue reactivations are predominantly not compressed (median of $2\times$; two-sample two-tailed Kolmogorov-Smirnov test; $p < 0.001$). **c** Same as **b** in boxplot (two-tailed Mann-Whitney U-test; $p < 0.001$). Source data are provided as a Source Data file.

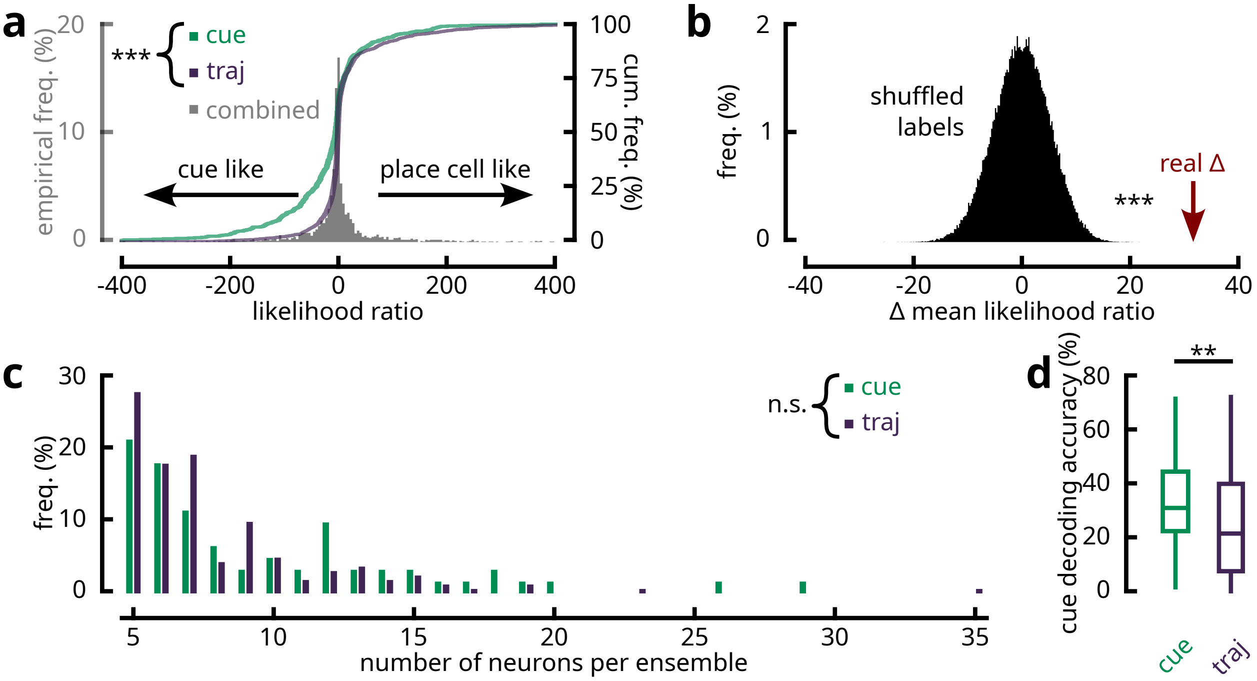

Supplementary Figure 10

Cue and trajectory information are conjunctively encoded by resting-state ensembles; however, ensembles express varying degrees of bias for each separate behavioural feature. a Two models were fitted to the activities of ensemble neurons (see Methods): one is driven by sensory cues and the other is modelled after a *place-cell*'s tuning curve. Taking the likelihood ratio between the two models gives a neuron's proclivity for either type of response tuning. The distribution of these ratios express no bimodality, suggesting that ensemble neurons encode for a conjunction of cue and place responses. Labelling the neurons based on their cue/trajectory ensemble membership showed however that ensembles are biased in the type of behavioural features their encode for ($n = 601$ cue ensemble neurons; $n = 1314$ trajectory ensemble neurons; two-sample Kolmogorov–Smirnov test $p < 0.001$). **b** Distribution of the difference between mean likelihood ratios between cue and trajectory ensemble neurons, obtained by permutating the membership labels. The real difference is identified by the red arrow, confirming the bias for cue and trajectory ensembles in encoding their respective features ($p < 0.001$). **c** Distribution for the number of neurons per cue or trajectory ensemble. Cue and trajectory ensembles supported a similar number of neurons (χ^2 test; $p = 0.17$), meaning that any detected differences in the encoding of behavioural parameters were not due to bias in sample size. **d** Bayesian decoding accuracy for the identity of individual cues using cue and trajectory ensemble neurons. Cue ensemble neurons were more accurate at identifying the identity of the four cues (two-tailed Mann-Whitney U-test; $p = 0.0012$), likely due to their tendency of supporting multiple place fields with different firing rates at different cue locations. However, this accuracy was still low at a median of $\sim$30 %. This could be due to the responses of cue ensemble neurons being targetted at specific cues rather than all four cues indiscriminately. Source data are provided as a Source Data file.

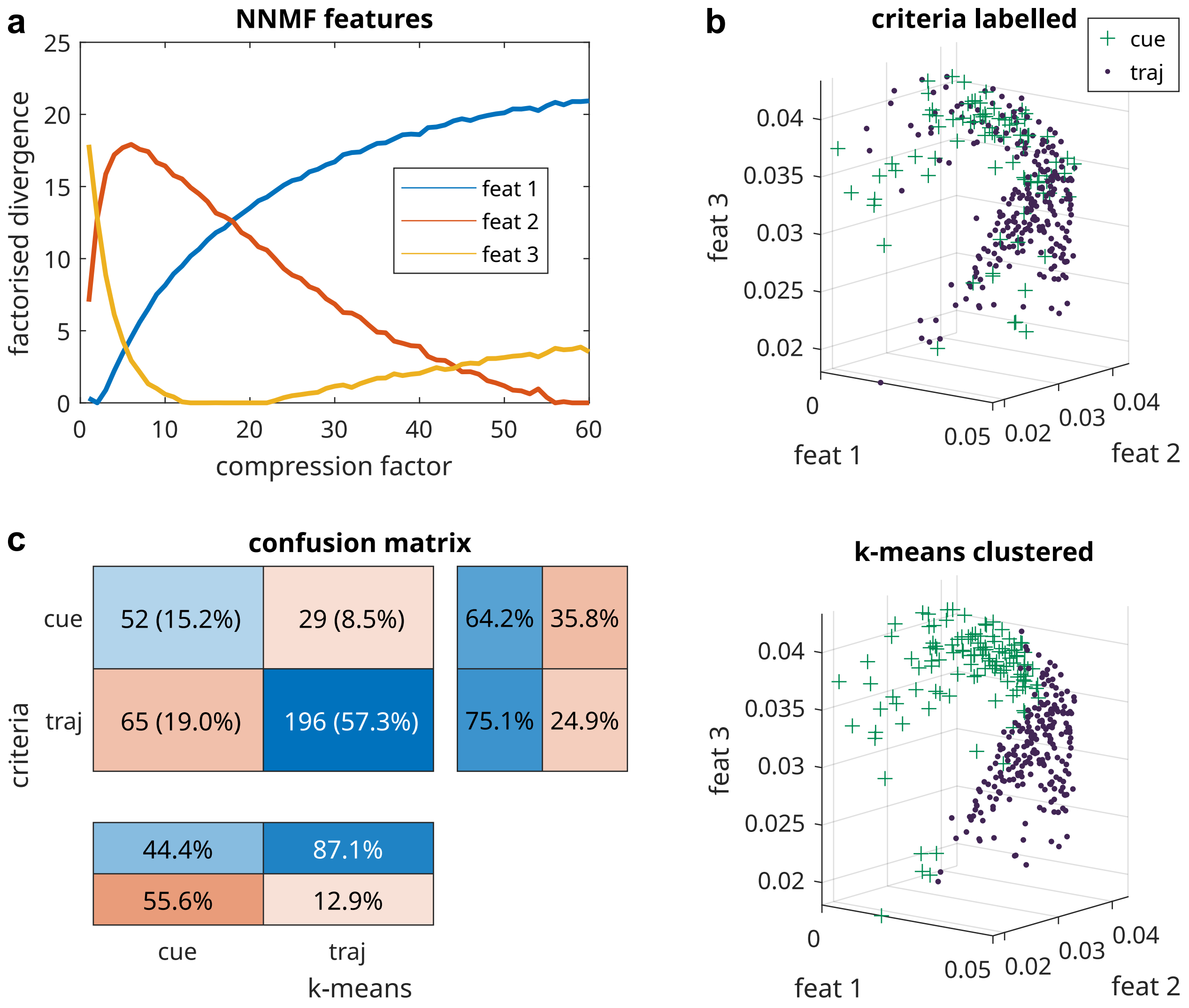

Supplementary Figure 11

Cue and trajectory ensembles emerge as separate categories under unsupervised clustering of temporal compression. **a** The three features extracted by NNMF are shown. Notice that feature 3 peaks in the beginning and sharply decreases with higher compression factors, closely resembling the trend observed for cue ensembles in Supplementary Fig. 9a. Similarly, feature 2 has a later peak and decays at a slower rate, following a similar pattern as trajectory ensembles. **b** Ensembles projected into the coordinates given by the coefficients matrix. Class labels are assigned either by the selection criteria defined in Methods or by k-means clustering. In accordance with our hypothesis, cue ensembles have higher weights over the axis of feature 3, which corroborate with their affinity to lower temporal compression. In contrast, trajectory ensembles have higher weights over feature 2, which reinforces the notion that trajectory reactivations are temporally compressed. Visually, the clusters isolated by the selection criteria are comparable to those identified by k-means. Note, however, that this point cloud forms a continuous density as opposed to two visually separate clusters, which corroborates with the notion that a continuum likely describes the affinity of ensembles for either cue or trajectory. **c** Confusion matrix comparing the labels assigned by criteria and the labels determined by k-means. The two labelling methods agreed strongly on the

identity of trajectory ensembles. Cue ensembles labelled under k-means were, however, less than half as likely to be also considered as cue ensembles under selection criteria. These are likely reactivation of short trajectory segments that fell outside of the cue zone, which NNMF and k-means are unable to distinguish due to not having knowledge of the locations of cues. Overall, cue and trajectory labels assigned by criteria and by unsupervised clustering show a significant degree of overlap (χ^2 test; $p < 0.001$; $\chi^2 = 42.405$; df = 1).

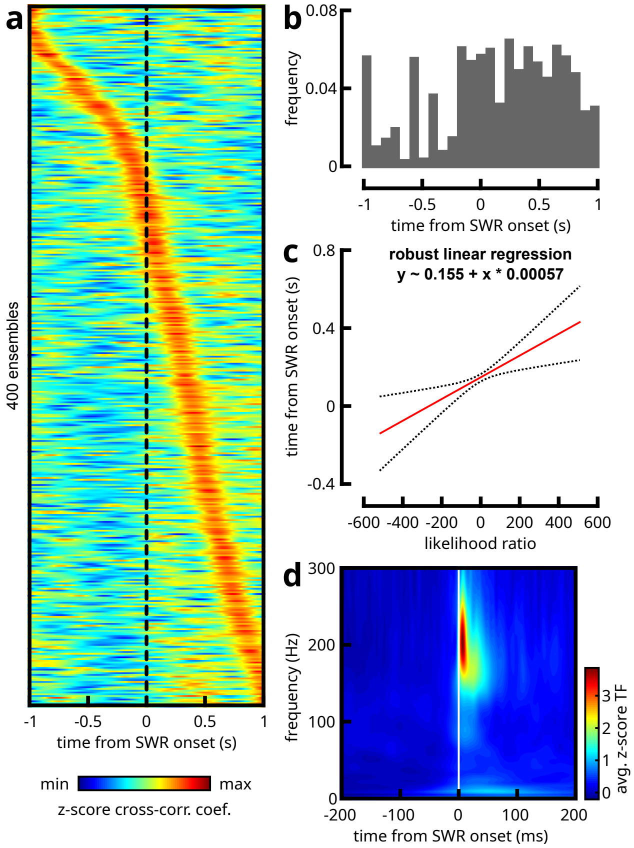

Supplementary Figure 12

Timing relationship between reactivation and SWR events is biased by cue versus position encoding. a Z-scored cross-correlogrammes (as in Fig. 4**f**) between SWR onset times and ensemble reactivation onset times. The majority of ensemble reactivations follow from SWRs. **b** Histogram of peak cross-correlation time lags in **a**. **c** A regression analysis was sought to model the relationship between cells' tendency for encoding either cue or positional features and the timing of their activity with hippocampal SWRs. From **b**, it is clear that the distribution of timings is not normal. To compensate for this limitation, a robust linear regression was conducted. The time lag of peak cross-correlation was modelled as a function of the likelihood ratio, which was used as a measure of cue versus place coding (see eq. 12). Only cells that were part of resting-state ensembles and that passed the criteria for spatial selectivity were considered ($n = 1309$ neurons), while the corresponding peak time lag was derived from the associated ensemble. The fitted model has an intercept at 0.155 s ($p < 0.001$) and a positive slope of 0.57 ms per unit of likelihood ratio ($p = 0.0043$; $R^2_{\mathrm{adj}} = 0.0034$). The fitted model is commensurate with the notion that more cue-tuned cells belong to ensembles that reactivate earlier in time in relation to SWRs, while this timing is progressively delayed as cells are more position-tuned. **d** Time-frequency plot obtained by continuous wavelet transform centred at the onset of SWRs in one example recording session. Source data are provided as a Source Data file.

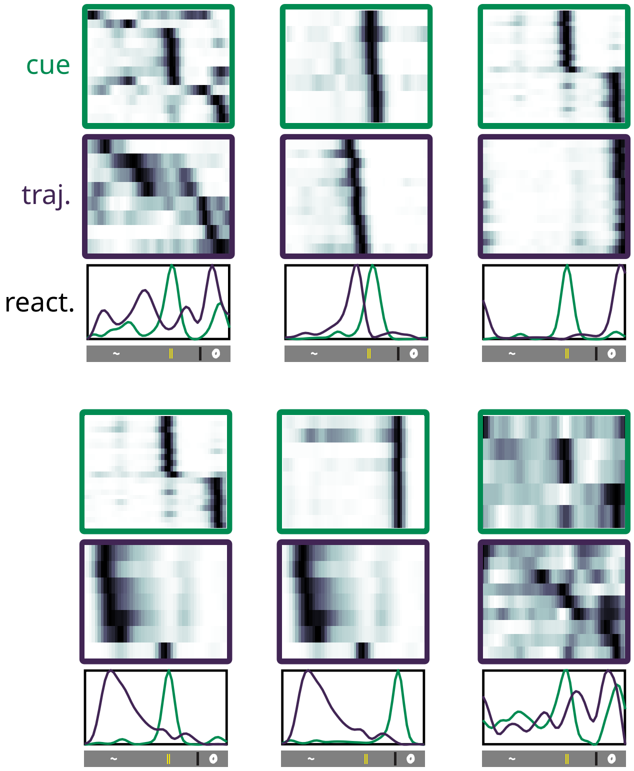

Supplementary Figure 13

Six examples of temporally coupled cue-trajectory ensemble pairs. The average neuronal activities as a function of spatial location for all neurons belonging to a cue or trajectory ensemble are shown. Neurons were sorted by location of peak activity. The corresponding reactivation strengths as a function of spatial location for the corresponding cue and trajectory ensembles are illustrated.

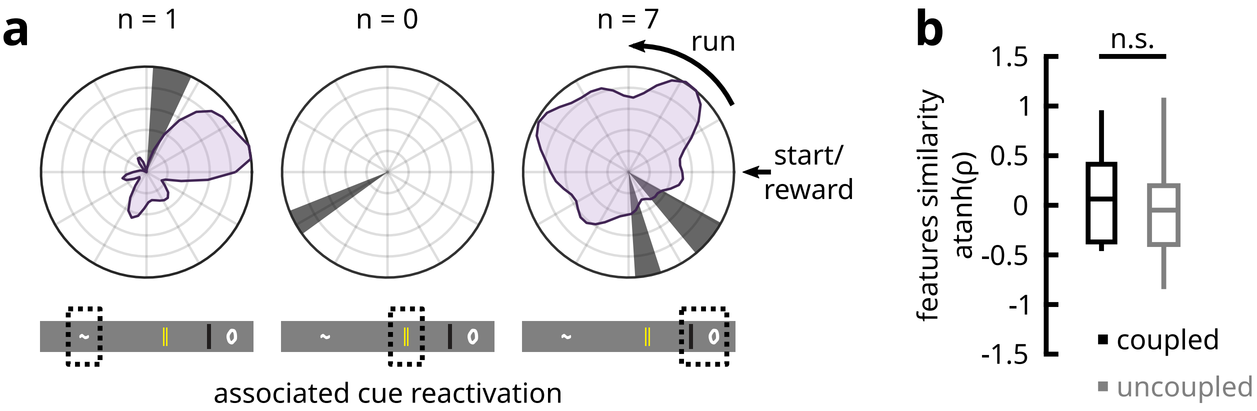

Supplementary Figure 14

Cue and trajectory ensemble pairs from REST1 that are temporally-coupled. **a** Same as Fig. 5**d** for coupled REST1 ensembles. Out of the eight pairs of cue and trajectory ensembles, seven were associated with the later two cues, while one is associated with the first cue. **b** Given the small sample size, the cue identify was omitted (cf. Fig. 5**e**), and only the main effect of temporal-coupling was tested. No significant difference in the similarity of reactivated features between coupled and uncoupled ensemble pairs was detected (two-tailed two-sample t-test on atanh-transformed Pearson correlation coefficients; $p = 0.494$). However, given the low statistical power owing to the restricted sample size, it cannot be concluded that REST1 ensembles do not reactivate for complementary features. Source data are provided as a Source Data file.

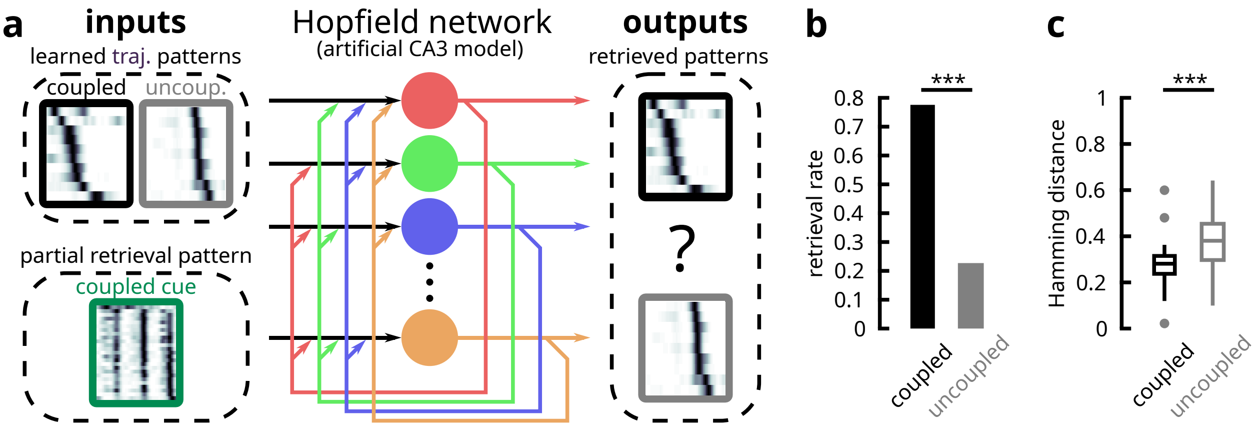

Supplementary Figure 15

Hopfield network modelling for the retrieval of trajectories by seeding with cue information. **a** Illustration of the Hopfield network model. Within each session where a 'coupled' pair was present, the reactivated trajectory features from the 'coupled' ensemble and from one 'uncoupled' ensemble were used as training patterns fed to the Hopfield network. The 'coupled' cue ensemble pattern was subsequently used as a partial/degraded pattern to retrieve one of the two stored patterns. **b** Temporally 'coupled' trajectory features were more likely to be retrieved from the Hopfield network by the associated cue features than the 'uncoupled' trajectory features (binomial test; $p < 0.001$). **c** The Hamming distances between the reactivated features of 'coupled' pairs were shorter than those of the 'uncoupled' pairs (one-tailed Mann-Whitney U test; $p < 0.001$), which again suggests a greater degree of similarity in reactivated features between temporally-coordinated cue/trajectory ensemble pairs. Source data are provided as a Source Data file.

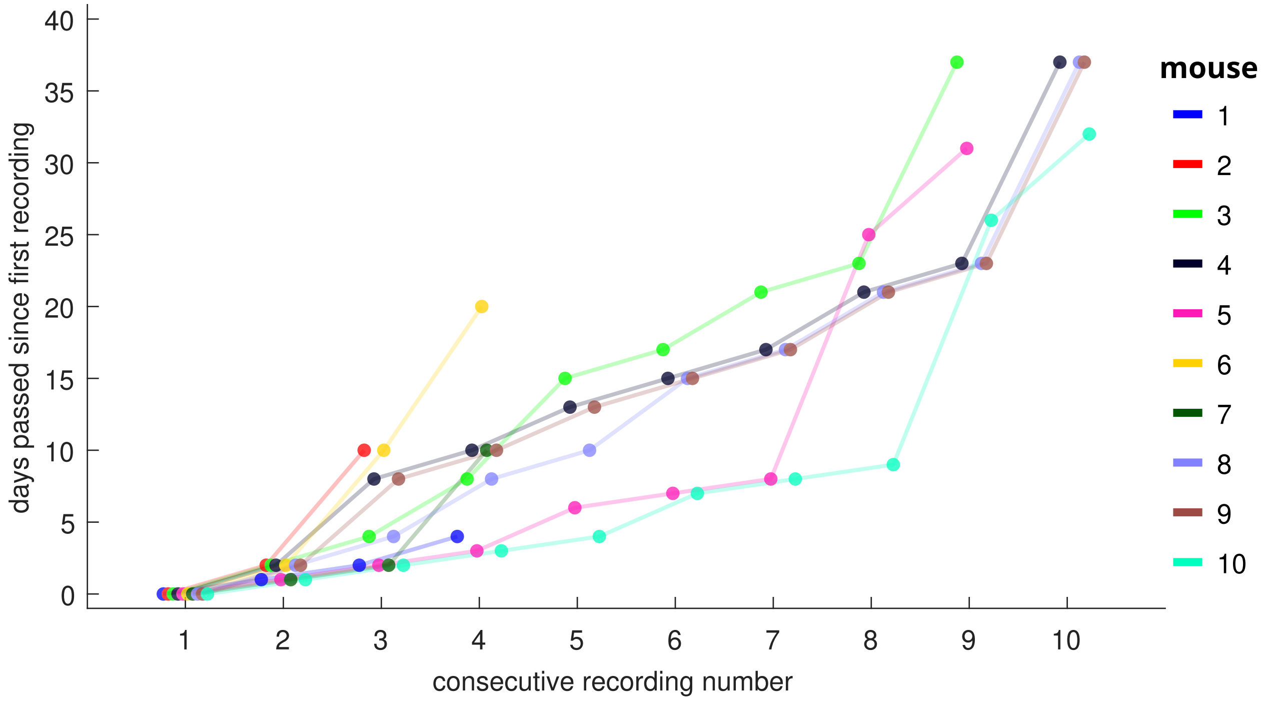

Supplementary Figure 16

The number of days elapsed since the first recording for each consecutive recording sessions in the ten animals in which the same neuron ROIs were tracked across time.

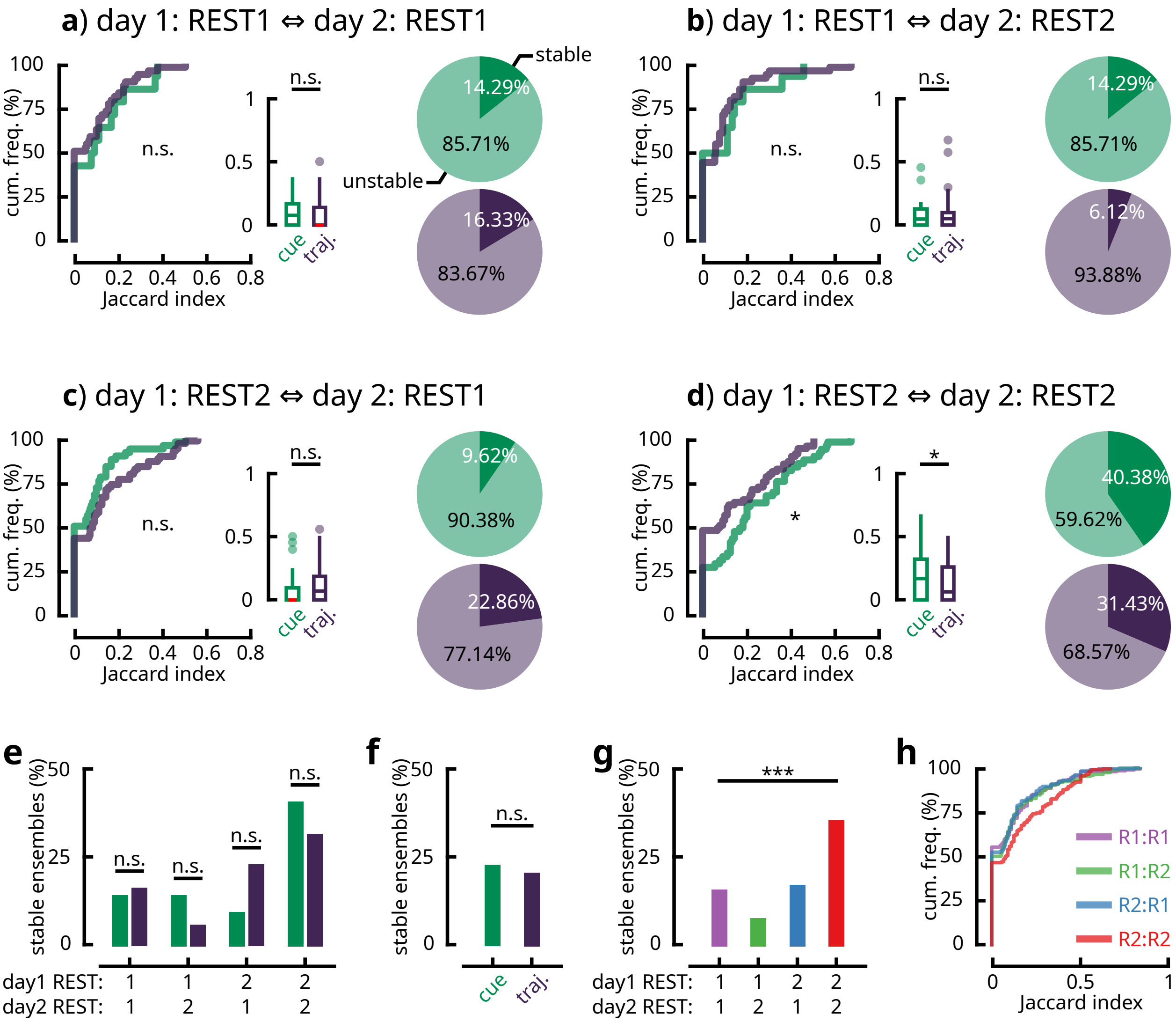

Supplementary Figure 17

Resting state ensembles observed following active locomotion persist across recording days. a-d The proportions of overlapping ensemble members (quantified as Jaccard distances) and the percentage of persistent ensembles across consecutive recording days were determined using the same procedures described in Fig. 6**e-f**. All combinations of REST1/REST2 ensembles across days were evaluated (e.g., in **a**, REST1 ensembles on the reference day were matched with REST1 ensembles on the subsequent recording day). Out of these combinations, cue ensembles ($n = 14$ in REST1; $n = 52$ in REST2) from REST2 on the reference day were slightly more stable than trajectory ensembles ($n = 49$ in REST1; $n = 70$ in REST2) in REST2 on the subsequent day (two-sample two-tailed Kolmogorov-Smirnov test $p = 0.024$; two-tailed Mann-Whitney U-test $p = 0.029$). Otherwise, no differences had been observed in the other combinations. Overall, a greater fraction of both cue and trajectory ensembles persisted in REST2 across recording days (**d**). **e** To further corroborate the results in **a-d**, pairwise χ^2 tests were performed between the proportions of persistent cue and trajectory ensembles over all REST combinations (df = 1; significance $\alpha = 0.05$). No differences between the fractions of stable cue and trajectory ensembles were found across all conditions, including in REST2-REST2, even prior to adjusting for multiple comparisons. There-

fore, the increased persistence of cue ensembles across days following locomotion, observed in **d**, was marginal. **f** At the group level (i.e., taking the sum across all REST combinations), there was no difference in persistence between cue and trajectory ensembles either ($\chi^2 = 0.231$; df = 1; $p = 0.631$). **g** There was however a main effect of across-days REST combinations on the stability of ensembles, without accounting for cue/trajectory labels ($\chi^2 = 23.148$; df = 3; $p < 0.001$). **h** Plotting the empirical cumulative distribution functions of Jaccard distance for all REST combinations revealed that the proportions of stable cue or trajectory ensembles in REST2-REST2 was markedly higher than in other combinations. Taken together, these results suggest that following locomotion, similar resting state ensembles tend to be recruited across days, although no preference is assigned to either cue or trajectory ensembles. Source data are provided as a Source Data file.

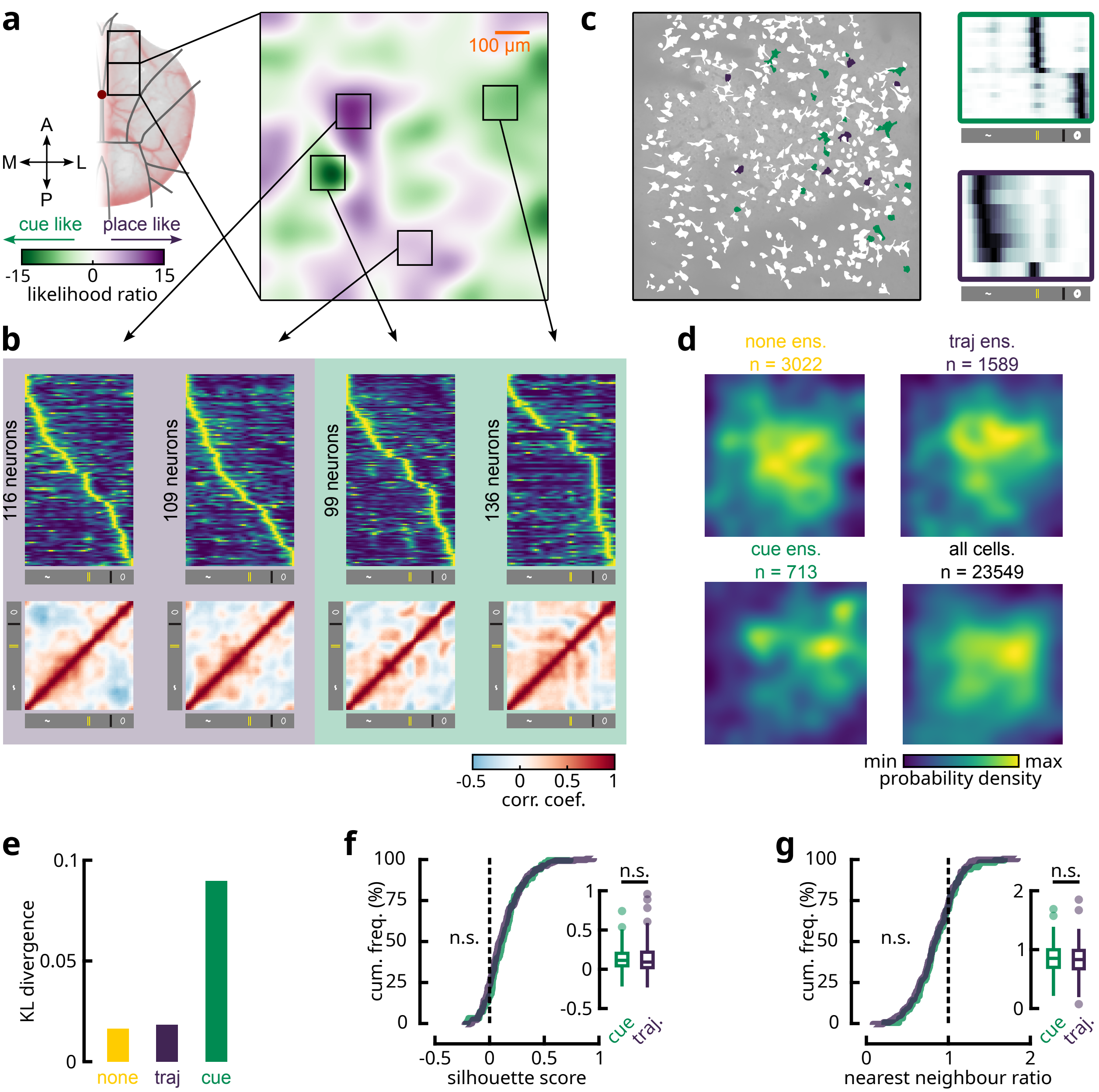

Supplementary Figure 18

Topographic organisation of cue and spatially-selective cells. a Topographic map of the distribution of cells' tendency for encoding either cues or spatial positions. **b** Four 100 µm × 100 µm windows were drawn over patches with higher densities of either cue or locations responses. The average responses over positions of the neurons, whose centroids fall within the boundaries of these windows, were obtained, along with the correlation matrices of the resulting population vectors. **c** Mask of neuronal ROIs in one example recording session. Individual examples of a cue and of a trajectory ensemble, and their corresponding ROIs are shown. **d** Density maps of the locations of neurons belonging to each resting ensemble class (REST1 and REST2 pooled), as well as all neuronal ROIs, were obtained by regular kernel density estimation using the same parameters as in **a**. **e** Kullback-Leibler divergence between the probability density maps of each ensemble class and the density map of all neuronal ROIs in **d**. **f-g** Averaged silhouette scores and

nearest neighbour ratios across neurons of a given ensemble ($n = 75$ cue ensembles; $n = 196$ trajectory ensembles). Overall, there was no difference in the degree of clustering between cue and trajectory ensemble neurons (two-sample two-tailed Kolmogorov-Smirnov test $p = 0.513$ for silhouette score, $p = 0.936$ for NNI; two-tailed Mann-Whitney U-test $p = 0.392$ for silhouette score, $p = 0.511$ for NNI). However, on both measures of clustering, over $\sim$70 % of ensemble neurons of either category tend to be more clustered in topographic space, rather than dispersed (Wilcoxon signed rank test $p < 0.001$ for median silhouette score equals 0, $p < 0.001$ for median NNI equals 1). Source data are provided as a Source Data file.

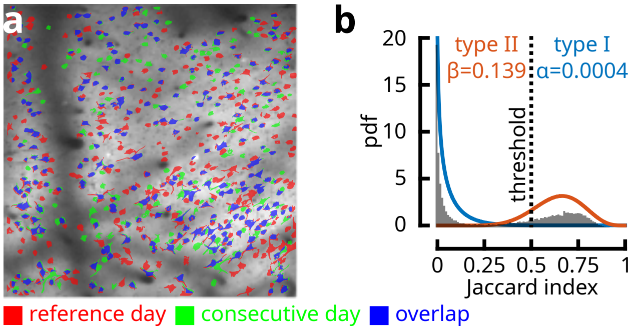

Supplementary Figure 19

Identification of the same neuronal ROIs across consecutive days of recording. **a** Registered ROI masks for neurons detected on the reference day (red) and on the subsequent day of recording (green), in one example session. Overlapping pixels are coloured in blue. **b** Probability density function of the percentage of overlap (measured by Jaccard distance) between neuron ROIs across days, for all neuron pairs with at least one overlapping pixel. The histogram reveals a bimodal distribution consisting of persistent and differing cells. These two clusters were separated by K-means and were each fitted to a beta distribution by maximum likelihood estimation. The beta distribution, commonly used to model percentages and probabilities, is defined over the interval $[0, 1]$ and aptly describes Jaccard distances. With the overlapping threshold set at 50 %, the left-side beta distribution (blue), which represents unstable neurons, estimates a false positive rate of 0.04 %. Meanwhile, the statistical power given by the right-side distribution (orange) is estimated at 86.1 %. Therefore, a 50 % overlapping threshold for distinguishing persistent neurons across days is a highly robust criterion for the present dataset, at a reasonable cost to statistical power. This model was inspired by [2]. Source data are provided as a Source Data file.

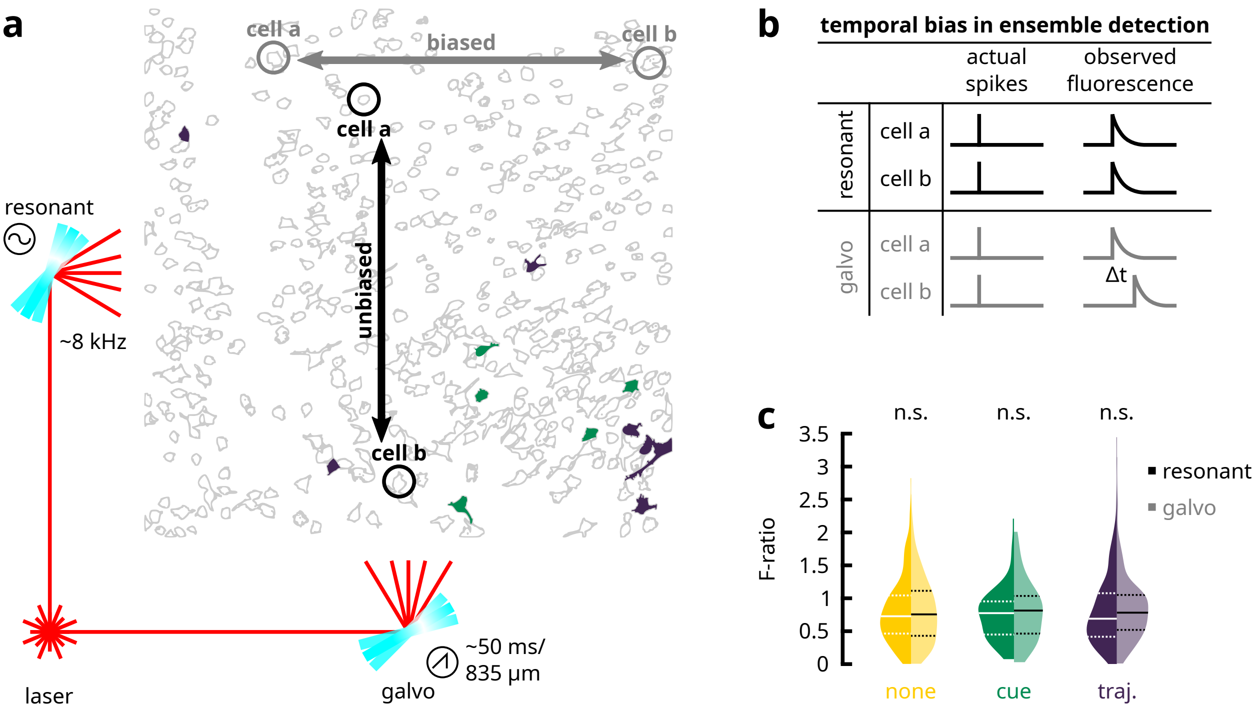

Supplementary Figure 20

Detection of offline ensembles is not biased by the scan mirrors. a The laser beam is directed to the two axes of the field-of-view by a galvanometer scan mirror and a resonant scan mirror respectively. Examples of two offline ensembles (a cue ensemble in green and a trajectory ensemble in purple) are superimposed over all detected ROIs. **b** A direct consequence of this preparation is that neurons that are farther apart over the galvo axis (up to the midway point between the scanning path and the mirror flyback) are susceptible to shifted/delayed temporal dynamics. This can introduce a bias in the detection of offline ensembles, where synchronous ensembles found along the resonant axis are more likely to be detected compared to synchronous neurons along the galvo axis. Temporal smoothing had been performed to compensate for this issue. **c** To verify that this bias did not impact the detection, the following statistical hypothesis was proposed: the variance in the positions of ensemble neurons along the galvo axis, normalised by the total variance of all ROIs (i.e., the F-ratio), should be equal to that along the resonant axis. This null hypothesis held for all detected offline ensembles, irrespective of category (paired-sample two-tailed Wilcoxon signed rank tests; $p = 0.5$ for none; $p = 0.36$ for cue; $p = 0.273$ for trajectory). Therefore, synchronous neuron pairs are as likely to be detected over the span of the resonant axis as they are over the galvo axis. Source data are provided as a Source Data file.

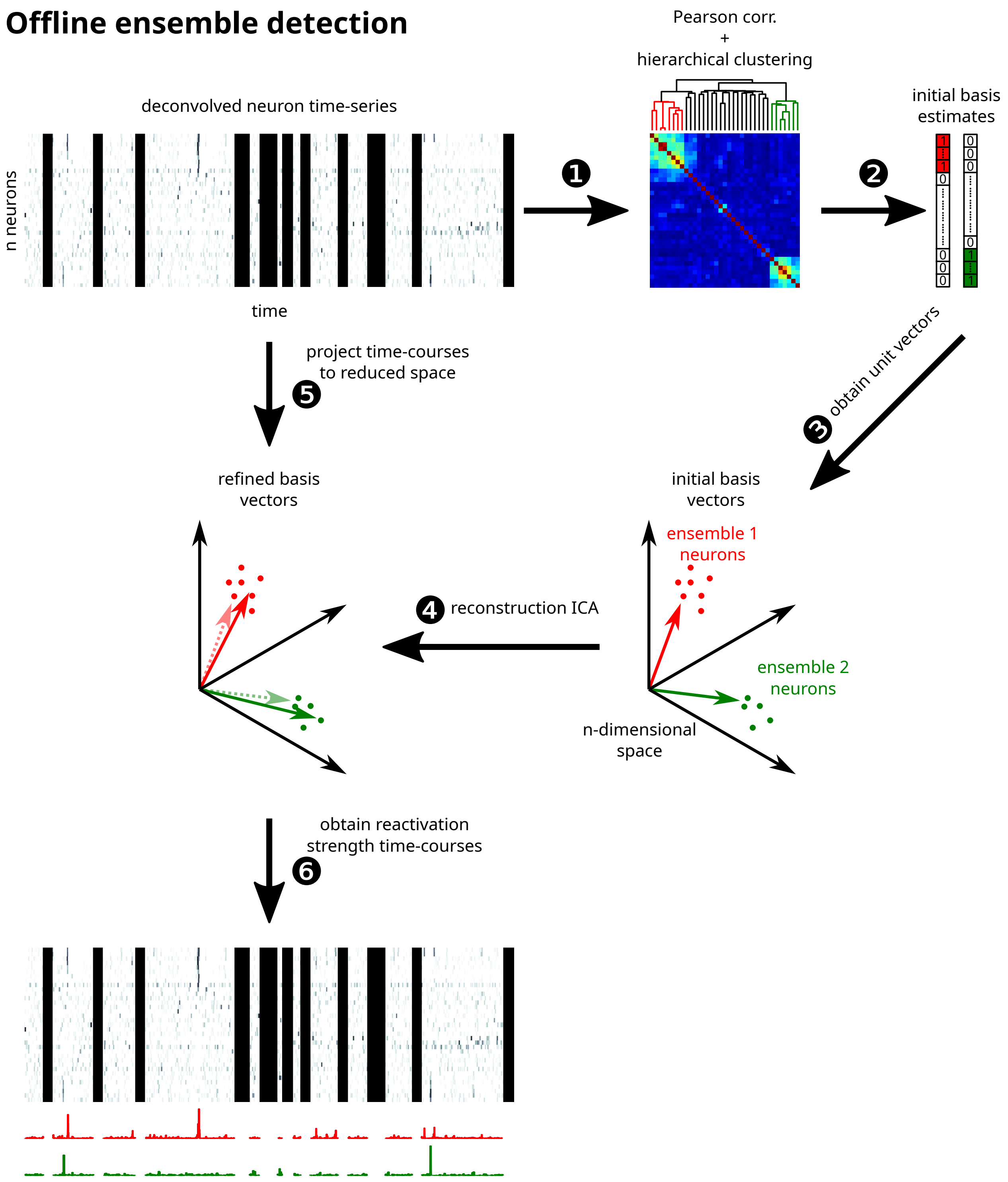

Supplementary Figure 21

Methods used for detecting synchronous resting-state ensembles and for establishing the time-courses of their reactivation. See Methods for detailed description. 1) Hierarchical clustering is conducted over the Pearson correlation matrix of the neuronal time-series for the resting period. 2) For each ensemble, a binary column vector of the same length as the total number of neurons is created. Members belonging to the ensemble are labelled as ‘1’, while the remaining

are ‘0’. 3) Normalizing these vectors by their norm yields a set of unit vectors that together form an orthonormal basis. 4) Using reconstruction ICA, these basis vectors are fine-tuned in such a way as to retain most of the variance in the original data, and in doing so capture the relative contributions/weights of each member neuron to an ensemble’s temporal dynamics. 5) The original neuronal time-series matrix is projected into the new space defined by the basis. 6) This projection yields the reactivation strength of each ensemble as a function of time.

Detection of reactivated online features

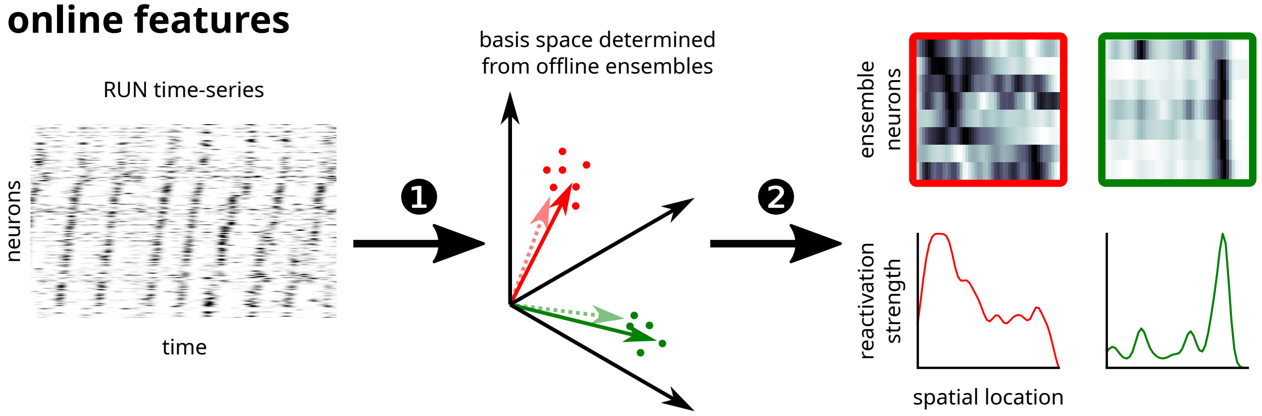

Supplementary Figure 22

By projecting the neuronal time-series matrix during RUN into the space defined by the basis estimated from resting network dynamics (1), the ensemble activation strength as a function of animal location can be obtained (2).

Supplementary Tables

				number of ensembles							
				REST1				REST2			
animal	num. sessions	LFP	ROI tracking	none	cue	traj.	all	none	cue	traj.	all
1	6	n/a	n/a	12	0	7	19	13	0	10	23
2	5	n/a	n/a	24	2	10	36	16	3	34	53
3	1	n/a	n/a	6	1	2	9	0	0	2	2
4	4	avail.	avail.	10	0	3	13	9	1	2	12
5	3	poor	avail.	19	1	13	33	16	8	13	37
6	1	avail.	n/a	4	0	1	5	6	1	3	10
7	9	avail.	avail.	19	1	4	24	38	1	15	54
8	10	avail.	avail.	30	5	9	44	12	17	12	41
9	9	avail.	avail.	26	0	4	30	13	4	3	20
10	4	avail.	avail.	14	3	8	25	26	6	16	48
11	4	poor	avail.	9	1	15	25	7	10	17	34
12	10	avail.	avail.	15	1	0	16	12	1	8	21
13	10	avail.	avail.	50	1	7	58	52	0	10	62
14	10	avail.	avail.	38	3	14	55	32	10	19	61
total	86	9	10	276	19	97	392	252	62	164	478

Supplementary Table 1

Experimental conditions for individual animal subjects.

Supplementary Methods

Estimating proportions of cue/spatial cells

Ideally, cells that respond exclusively to visuo-tactile cues would have been identified by introducing laps over a blank belt and comparing the responses of neurons following the addition of cues. This was, however, not the case in the current set of experiments. Therefore, only a rough estimation of the proportions of cue-responsive cells can be made. For this estimation, we assume that cortical spatial cells are uniformly scattered over locations, consistent with the behaviour of hippocampal place cells (cf. [3]). At the locations of cues, extra cells will be found responding to the sensory cues. Therefore, if we obtain the locations at which neurons show peak firing, the resulting distribution is expected to be approximately uniform, with elevated density at the locations of cues (Supplementary Fig. 7**a-b**).

It follows that, if we are to iteratively remove neurons whose peak responses occurred at cue locations, then the distribution of activity peaks will gradually approach a uniform distribution. We used the χ^2 statistic to measure the uniformity of this distribution:

$$\chi^2 = \sum_{i=1}^{n} \frac{(O_i - \frac{N}{n})^2}{\frac{N}{n}}, \tag{1}$$

where O_i is the number of peaks in bin i, for which there are $n = 50$ bins and N peaks in total. $\frac{N}{n}$ is the expected number of peaks at each location under the assumption of uniformity. The objective is to minimize χ^2 by removing peaks falling within the locations of cues (i.e., to fit parameters O_i for all i bins coinciding cue locations and adjusting N accordingly). We solved this minimization problem by the interior-point method. Finally, taking the fraction between the number of cells removed following "uniformization" of the distribution of peaks and the total number of cell yields the estimated proportion of cue-responsive cells (Supplementary Fig. 7**c**).

In the first and last bins, an unusually elevated number of peaks are found, likely reflecting cells responding to reward (Supplementary Fig. 7**a-b**). These bins were omitted from the optimization.

Temporal Compression

Given the low sampling rate of our imaging data, estimating the temporal compression during replay events may not proceed in the same manner as detailed in [4] (i.e., template matching) without the risk of incurring a substantial bias. This is because the median duration of reactivation events is on the orders of ∼200 ms (Fig. 4**g**), which corresponds to only 4 imaging frames, too few to accurately represent any putative sequences in the reactivation events. Instead, a surrogate analysis was derived from our PCA-ICA method. Following on Equation 6, we extracted the principal components corresponding to each resting-state ensemble. We reasoned that, by downsampling (i.e., compressing) the RUN time-series matrix, these resting-state principal components would explain a higher fraction of the variance in the RUN data, if the RUN data corresponding to the rest ensembles is organised in a sequential form. In other words, if a component captures a trajectory ensemble, then temporal compression of the RUN time-series would lead to the sequence collapsing into synchronous activities similar to those observed during rest, from which the component was derived. In contrast, cue ensembles should not benefit from such compression, as the neurons

would already fire in synchrony during the RUN periods in response to cue sensations. To quantify this relationship, we proposed a measure of *divergence*, given as

$$d = 1 - \left|\frac{r^2_{\mathrm{RUN}}}{r^2_{\mathrm{REST}}} - 1\right|, \tag{2}$$

where r^2_{RUN} and r^2_{REST} correspond to the proportion of variance explained by a given PCA-ICA component during RUN and REST, respectively. This measure has an upper bound of 1, where a value close to 1 indicates that the fraction of variance explained by a given component is similar between RUN and REST behavioural states. This divergence was calculated at each compression of the RUN time-series. Downsampling was conducted using an averaging filter of the same order as the compression factor.

To further validate our criteria for the classification of cue and trajectory ensembles, we sought an unsupervised clustering method that is not biased by assumptions. The temporal compression analysis (Supplementary Fig. 9) provides an elegant way to discover these putative ensemble classes; if cue and trajectory ensembles are distinguished by the presence of a sequential structure, then in principle they should organise into two separate clusters in a feature space that describes their affinity to temporal compression. To test this hypothesis, *non-negative matrix factorisation* (NNMF) was performed over the divergence matrix of ensembles (a $c \times e$ matrix where the rows correspond to different compression factors, the columns to individual ensembles and the values are the divergence scores reported in Supplementary Fig. 9). Note that, though our divergence measure does not have a lower bound, no negative values were obtained in practice. NNMF factorises the divergence matrix into two matrices. The first matrix (the 'feature' matrix) extracts an arbitrary number of components that accounts for regular patterns in the divergence measures across all ensembles. The second matrix (the 'coefficients' matrix) attributes weights to individual ensemble for each of these distinctive features. NNMF was conducted using three components, in consideration of the three putative ensemble classes proposed (none, cue and trajectory).

Modelling cue- and position-correlated responses

In order to quantify the individual contributions of visuo-tactile cues and spatial locations to neuronal responses, two candidate models were fitted to the activity time-courses of neurons. In the first model, which aims to reflect the individual neuronal responses that form a trajectory sequence, it was assumed that the firing rate profile of trajectory ensemble neurons follow that of 'place cells'. Accordingly, a Gaussian function was used to describe a neuron's firing rate as a function of location:

$$\lambda(x) = A\exp\left(-\frac{(x-B)^2}{2C^2}\right). \tag{3}$$

Here, the neuron's activity as a function of position $\lambda(x)$ follows a Gaussian function with amplitude A, place field centre B and standard deviation C. Parameters A through C were fitted by maximizing the likelihood function formulated below (eq. 5) using an *interior-point* approach. The optimization was subjected to the following constraints: $A \geq 0$ and $0 \leq B \leq 150$ (corresponding to the length of the belt).

In the second model for cue responses, the mean activity rates $(\lambda_1, \ldots, \lambda_4)$ in each individual space defined by visuo-tactile cues (a, b, c, d), along with the baseline activity outside of cue

locations (λ_0), were obtained such that the position-dependent response function is given by:

$$\lambda(x) = \begin{cases} \lambda_1 & \text{if} x \in a \\ \lambda_2 & \text{if} x \in b \\ \lambda_3 & \text{if} x \in c \\ \lambda_4 & \text{if} x \in d \\ \lambda_0 & \text{if} x \notin a \cup b \cup c \cup d \end{cases} . \tag{4}$$

As such, neurons can support multiple place fields in the form of a distinct firing rate at each unique cue location. The quality of the fitted models at explaining the observed neuronal responses was assessed from a likelihood function. Assuming that neurons' activity patterns follow an inhomogeneous Poisson process, it can be shown that the log-likelihood of a candidate model given the activity and position vectors from the observed data is expressed as:

$$l(\lambda; n_1, \ldots, n_T, x_1, \ldots, x_T) = \sum_{i=1}^{T} n_i \log(\lambda(x_i)) - \lambda(x_i) - \log(\Gamma(n_i + 1)). \tag{5}$$

Here, the time-course vectors of neuronal activity and animal position are represented by n_i and x_i respectively, and the activity rate function $\lambda(\cdot)$ is given by one of the candidate models. It is important to note that it is inaccurate to describe neuronal responses, as observed from calcium fluorescent transients, through a Poisson distribution, given that the sampling rate of the technique is too slow to discern individual spikes. However, it has been shown previously that this assumption, as a first-order approximation, does model accurately the relationships between calcium activity and behavioural parameters [1,5]. In the present scenario, where the time-course vector n_i resides over a positive continuous domain, the integer factorial was substituted for the gamma function following the relationship $\Gamma(n+1) = n!$.

The likelihood ratio was taken between the likelihoods under the two candidate models to evaluate which of the two models is better supported by the observed data:

$$L_{\text{R}} = -2(l_{\text{cue}} - l_{\text{place}}). \tag{6}$$

Under this ratio, higher values imply that the 'place cell' model is a stronger candidate than the 'cue' model and *vice versa* for lower values.

Hopfield network modelling

The reactivated features (i.e., the reactivation strength as a function of spatial position) were discretized into 50 spatial bins and normalized between 0 to 1. These vectors were then binarized at a threshold of 0.5, where bins with activity higher than 0.5 are set to +1 and the remaining are set to -1. At the learning stage, a weight matrix $w_{i,j}$ is constructed from a pair of trajectory patterns that are to be stored by the Hopfield network (cf. [6]):

$$w_{i,j} = \frac{1}{2} \sum_{p=1}^{2} v_i^p v_j^p, \tag{7}$$

where v_i^p is the value of the pattern p in bin i, and the diagonal of the weight matrix is set to 0. At the retrieval stage, the retrieval cue pattern s_j with $N = 50$ spatial bins is presented:

$$s_j(t+1) = \text{sgn}(\sum_j^N w_{i,j} s_j(t)), \tag{8}$$

where t is the iteration number and $\text{sgn}(\cdot)$ is the sign function (turning s back into a binary vector with values of +1 and -1). Equation 8 is repeated until one of the two stored trajectory patterns is retrieved or when the number of iterations exceeds 50 in which case the trial is considered to have failed to converge.

Topographic Organisation

Out of 86 imaging sessions ($n = 14$ mice), 74 sessions ($n = 11$ mice) were conducted over the posterior window. Given these unequal sample sizes, the current topographic analysis will only focus on the posterior aspect of the secondary motor cortex, in consideration for adequate statistical power. A weighted kernel density estimation procedure [7] was used to construct a topographic map of the distribution of cells' tendency for encoding cues versus spatial positions. First, the centroid coordinates of neuronal ROIs were obtained and pooled across all animals and sessions. The likelihood ratios for each neuron, that passed the criteria for spatial selectivity, were then calculated for each centroid following Equation 12 ($n = 7871$ neurons). A weighted average of the likelihood ratio was then computed at each topographic bin (400×400 bins), where the weights are assigned by a Gaussian kernel with a bandwidth of 50 µm.

Two measures of clustering/dispersion in topographic space were computed. On the one hand, the silhouette score is given as

$$s_i = \frac{b_i - a_i}{\max\{a_i, b_i\}}, \tag{9}$$

where, for a given ensemble neuron i, a_i is the mean Euclidean distance between it and all other neurons belonging to the same ensemble, and b_i is its mean distance from all other neurons that are not part of the ensemble. The silhouette score ranges between -1 and 1, with higher positive values corresponding to higher clustering and lower negative values signifying more dispersion.

On the other hand, the nearest neighbour ratio (NNI) was computed as

$$\text{NNI} = \frac{D_o}{D_e}, \tag{10}$$

where D_o is the average Euclidean distance between the nearest neighbours amongst neurons of a given ensemble, while D_e is the expected mean distance under random sampling. D_e was obtained by randomly drawing a sample of the same size as the number of neurons within the ensemble from all the available ROIs of a recording session. This random sampling procedure was performed 1000 times and the average was taken as the value of D_e. NNI values smaller than 1 tend towards clustering, while values greater than 1 suggest dispersion.

Supplementary References

[1] Esteves, I. M. *et al.* Spatial information encoding across multiple neocortical regions depends on an intact hippocampus. *The Journal of Neuroscience* **41**, 307–319 (2021).

[2] Sheintuch, L. *et al.* Tracking the same neurons across multiple days in ca2+ imaging data. *Cell reports* **21**, 1102–1115 (2017).

[3] Rich, P. D., Liaw, H.-P. & Lee, A. K. Large environments reveal the statistical structure governing hippocampal representations. *Science* **345**, 814–817 (2014).

[4] Euston, D. R., Tatsuno, M. & McNaughton, B. L. Fast-forward playback of recent memory sequences in prefrontal cortex during sleep. *Science* **318**, 1147–1150 (2007).

[5] Mao, D. *et al.* Hippocampus-dependent emergence of spatial sequence coding in retrosplenial cortex. *Proceedings of the National Academy of Sciences* **115**, 8015–8018 (2018).

[6] Hopfield, J. J. Neural networks and physical systems with emergent collective computational abilities. *Proceedings of the National Academy of Sciences* **79**, 2554–2558 (1982).

[7] Wolters, M. A. & Braun, W. J. A practical implementation of weighted kernel density estimation for handling shape constraints. *Stat* **7**, e202 (2018).
