# Supplementary material for: Cortical reactivation of spatial and non-spatial features coordinates with hippocampus to form a memory dialogue
Source: Nat Commun. 2023 Nov 27;14:7748. doi: 10.1038/s41467-023-43254-7 (PMC10682454; doi:10.1038/s41467-023-43254-7)
Supplement: Supplementary file 3 — Reporting Summary [file 41467_2023_43254_MOESM3_ESM.pdf]

nature portfolio

Corresponding author(s): HaoRan Chang

Last updated by author(s): Oct 22, 2023

# Reporting Summary

Nature Portfolio wishes to improve the reproducibility of the work that we publish. This form provides structure for consistency and transparency in reporting. For further information on Nature Portfolio policies, see our Editorial Policies and the Editorial Policy Checklist.

## Statistics

For all statistical analyses, confirm that the following items are present in the figure legend, table legend, main text, or Methods section.

| n/a | Confirmed | |
|---|---|---|
| ☐ | ☒ | The exact sample size (*n*) for each experimental group/condition, given as a discrete number and unit of measurement |
| ☐ | ☒ | A statement on whether measurements were taken from distinct samples or whether the same sample was measured repeatedly |
| ☐ | ☒ | The statistical test(s) used AND whether they are one- or two-sided<br>*Only common tests should be described solely by name; describe more complex techniques in the Methods section.* |
| ☐ | ☒ | A description of all covariates tested |
| ☐ | ☒ | A description of any assumptions or corrections, such as tests of normality and adjustment for multiple comparisons |
| ☐ | ☒ | A full description of the statistical parameters including central tendency (e.g. means) or other basic estimates (e.g. regression coefficient) AND variation (e.g. standard deviation) or associated estimates of uncertainty (e.g. confidence intervals) |
| ☐ | ☒ | For null hypothesis testing, the test statistic (e.g. *F*, *t*, *r*) with confidence intervals, effect sizes, degrees of freedom and *P* value noted<br>*Give P values as exact values whenever suitable.* |
| ☐ | ☒ | For Bayesian analysis, information on the choice of priors and Markov chain Monte Carlo settings |
| ☐ | ☒ | For hierarchical and complex designs, identification of the appropriate level for tests and full reporting of outcomes |
| ☐ | ☒ | Estimates of effect sizes (e.g. Cohen's *d*, Pearson's *r*), indicating how they were calculated |

*Our web collection on statistics for biologists contains articles on many of the points above.*

## Software and code

Policy information about availability of computer code

| | |
|---|---|
| Data collection | Behaviour and local-field potential were collected using the Axon pCLAMP 10 acquisition software. Microscope control and two-photon imaging were conducted using the ThorImage 4.1 software. |
| Data analysis | ROI detection was achieved using Suite2p (https://github.com/MouseLand/suite2p). Fluorescent traces were deconvolved by OASIS (https://github.com/j-friedrich/OASIS). The 'WRS' R package was used for robust statistical modelling. The code that supports the findings of the present study is available on GitHub at https://github.com/LelouchLamperougeVI/OfflineEnsembles and deposited in the Zenodo database under the accession code https://doi.org/10.5281/zenodo.10030861. |

For manuscripts utilizing custom algorithms or software that are central to the research but not yet described in published literature, software must be made available to editors and reviewers. We strongly encourage code deposition in a community repository (e.g. GitHub). See the Nature Portfolio guidelines for submitting code & software for further information.

## Data

Policy information about availability of data

All manuscripts must include a data availability statement. This statement should provide the following information, where applicable:

- Accession codes, unique identifiers, or web links for publicly available datasets
- A description of any restrictions on data availability
- For clinical datasets or third party data, please ensure that the statement adheres to our policy

Boundaries between cortical areas were determined from the Allen Common Coordinate Framework atlas (CCF v3). The data generated in this study have been deposited in the DRYAD database under accession code https://doi.org/10.5061/dryad.1ns1rn91c. Source data are provided with this paper.

## Human research participants

Policy information about studies involving human research participants and Sex and Gender in Research.

| | |
|---|---|
| Reporting on sex and gender | N/A |
| Population characteristics | N/A |
| Recruitment | N/A |
| Ethics oversight | N/A |

Note that full information on the approval of the study protocol must also be provided in the manuscript.

# Field-specific reporting

Please select the one below that is the best fit for your research. If you are not sure, read the appropriate sections before making your selection.

☒ Life sciences ☐ Behavioural & social sciences ☐ Ecological, evolutionary & environmental sciences

For a reference copy of the document with all sections, see nature.com/documents/nr-reporting-summary-flat.pdf

# Life sciences study design

All studies must disclose on these points even when the disclosure is negative.

| | |
|---|---|
| Sample size | No statistical methods were used to predetermine sample sizes, but our sample size of fourteen is on the upper end of those reported in previous publications of similar nature (e.g., Malvache et al. 2016, Sugden et al. 2020, Grosmark et al. 2021). In fact, studies typically employ 3-5 mice/rats to reliably observe reactivation events. We decided to slightly inflate our sample size in order to reliably detect the rarer reactivation of non-spatial cue representations. |
| Data exclusions | Of the eleven animals implanted with LFP electrode, two were excluded due to poor signal quality, likely caused by issues with grounding. These exclusions were made on the basis of visual inspection, with a prominent 60 Hz noise and large amplitude fluctuations coupled with animal movement serving as clear indications. |
| Replication | Reactivations have been observed in all animals (n = 14 mice). Important conclusions were verified using different and independent analyses and statistical models: 1. The presence of reactivation was confirmed by hierarchical clustering analysis as well as explained variance; 2. The presence of spatial and non-spatial reactivationing ensembles was shown using a thresholding method as well as likelihood modelling of custom probability distributions; 3. The temporal delay between cue and trajectory reactivations was confirmed by three independent cross-correlation analyses; 4. Feature similarities between time-locked cue and trajectory reactivationing ensembles were shown by Pearson correlation and Hopfield network modelling; 5. Persistence of ensembles across days were assessed by Jaccard distance as well as Chi-squared models (here, a slight discrepancy was found and discussed in the main text). |
| Randomization | Randomization and chance-level modelling procedures were introduced where biases may apply. Such includes the suffling procedure for the detection of position-correlated cells, auto/cross-correlation analyses, likelihood modelling and the dwell time of the laser over the pattern generated by the scan mirrors. |
| Blinding | This study had only a single experimental group, while reactivations, the type of information reactivated and sharp-wave ripples are all spontaneous events outside of the control of the experimenter. All data were processed equally by the same analysis pipeline. Therefore, blinding was not relevant. |

# Reporting for specific materials, systems and methods

We require information from authors about some types of materials, experimental systems and methods used in many studies. Here, indicate whether each material, system or method listed is relevant to your study. If you are not sure if a list item applies to your research, read the appropriate section before selecting a response.

## Materials & experimental systems

| n/a | Involved in the study |
|---|---|
| ☒ | ☐ Antibodies |
| ☒ | ☐ Eukaryotic cell lines |
| ☒ | ☐ Palaeontology and archaeology |
| ☐ | ☒ Animals and other organisms |
| ☒ | ☐ Clinical data |
| ☒ | ☐ Dual use research of concern |

## Methods

| n/a | Involved in the study |
|---|---|
| ☒ | ☐ ChIP-seq |
| ☒ | ☐ Flow cytometry |
| ☒ | ☐ MRI-based neuroimaging |

## Animals and other research organisms

Policy information about studies involving animals; ARRIVE guidelines recommended for reporting animal research, and Sex and Gender in Research

| | |
|---|---|
| Laboratory animals | Thy1-GCaMP6s mice between 2-8 months of age. Mice were single-housed under a 12 h light/dark cycle. The room temperature is kept at 22 degrees Celsius and the humidity fluctuates between 40 and 60% depending on the season. |
| Wild animals | This study did not involve wild animals. |
| Reporting on sex | Only male mice were employed in the present study. |
| Field-collected samples | This study did not involve samples collected from the field. |
| Ethics oversight | The care and use of animals in this study comply with the guidelines established by the Canadian Council on Animal Care and were approved by the Animal Welfare Committee at the University of Lethbridge. |

Note that full information on the approval of the study protocol must also be provided in the manuscript.
